# A Single Dose of Viperfav^TM^ May Be Inadequate for *Vipera ammodytes* Snake Bite: A Case Report and Pharmacokinetic Evaluation

**DOI:** 10.3390/toxins8080244

**Published:** 2016-08-19

**Authors:** Tihana Kurtović, Miran Brvar, Damjan Grenc, Maja Lang Balija, Igor Križaj, Beata Halassy

**Affiliations:** 1 Centre for Research and Knowledge Transfer in Biotechnology, University of Zagreb, Rockefellerova 10, HR-10000 Zagreb, Croatia; tkurtovi@unizg.hr (T.K.); beata.halassy@unizg.hr (B.H.); maja.langbalija@gmail.com (M.L.B.); 2 Centre for Clinical Toxicology and Pharmacology, University Medical Centre Ljubljana, Zaloška cesta 7, 1000 Ljubljana, Slovenia; damjan.grenc@kclj.si; 3 Institute of Pathophysiology, Faculty of Medicine, University of Ljubljana, Zaloška cesta 4, 1000 Ljubljana, Slovenia; 4 Department of Molecular and Biomedical Sciences, Jožef Stefan Institute, Jamova 39, 1000 Ljubljana, Slovenia; igor.krizaj@ijs.si; 5 Faculty of Chemistry and Chemical Technology, University of Ljubljana, Večna pot 113, 1000 Ljubljana, Slovenia

**Keywords:** *Vipera ammodytes ammodytes* envenomation, Viperfav™ antivenom treatment, F(ab’)_2_ antivenom pharmacokinetics

## Abstract

Viperfav^TM^ is a commercial F(ab’)_2_ antivenom prepared against European vipers venom. It is safe and effective for treating envenomation caused by *Vipera aspis* and *Vipera berus*. Therapeutic efficacy for treating *Vipera ammodytes ammodytes (V. a. ammodytes)* envenoming has not been yet described, although protective efficacy has been demonstrated in preclinical studies. We report on a 32-year-old man bitten by *V. a. ammodytes* who was treated with Viperfav™. Viperfav™ promptly reduced local extension and improved systemic pathological signs, but 24 h after the incident a recurrence of thrombocytopenia occurred despite a favorable pharmacokinetic profile with systemic clearance (1.64 (mL·h^−1^)·kg^−1^) and elimination half-life (97 h) among the highest ever reported. The recommended dose of Viperfav™ for *V. aspis* and *V. berus* bites may be inadequate for serious *V. a. ammodytes* envenomations. Following *V. a. ammodytes* bite, serial blood counts and coagulation profiles should be performed to help guide Viperfav™ treatment, along with supplemental administration as indicated.

## 1. Introduction

*Vipera ammodytes ammodytes* (*V. a. ammodytes)*, the nose-horned viper, is the most common snake of medical importance in Slovenia [1]. Snakebite victims have recently been treated with Viperfav™ (Aventis Pasteur, MSD, Lyon, France), a formulation containing polyvalent equine F(ab’)_2_ fragments as an active principle against *V. aspis*, *V. berus* and *V. a. ammodytes*. Viperfav™ has been proven to be safe and effective for rapidly counteracting the pathophysiological manifestations that follow *V. aspis* and *V. berus* bites [2,3,4,5,6,7]. Pharmacokinetic and therapeutic efficacy against *V. a. ammodytes* venom-induced toxicity in human has not been described, although neutralisation efficacy has been proven preclinically. We now report on a patient with *V. a. ammodytes* snakebite treated with Viperfav^TM^ in whom the pharmacokinetics of antivenom immunoglobulin F(ab’)_2_ fragments was performed.

## 2. Case Description

### 2.1. Case Report

**Figure 1:**
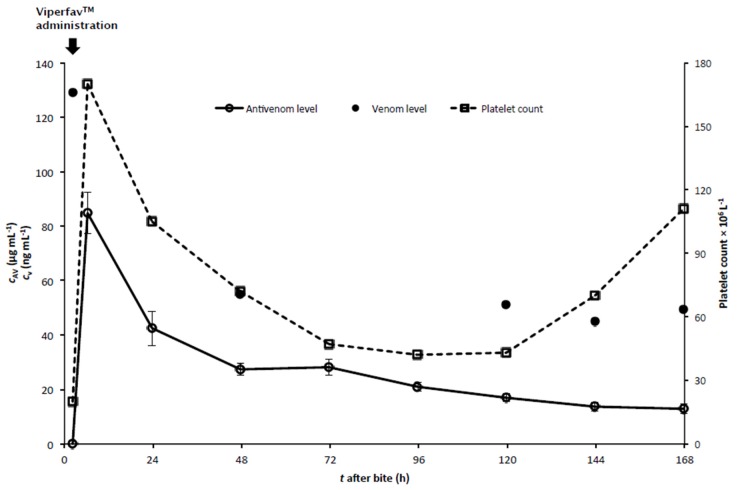
Platelet count and serum *V. a. ammodytes* venom (*c*_V_) and equine immunoglobulin F(ab’)_2_ fragments (*c*_AV_) concentrations in the patient bitten by *V. a. ammodytes* and treated with two vials of Viperfav™. Error bars represent 95% confidennce interval (CI) (*n* = 5).

A 32-year-old man with no previous medical history was gathering mushrooms in a wooded area in central Slovenia when he was bitten in the proximal phalanx of the fourth finger of the left hand by an approximately 60 cm long snake with a “horn” on the snout and a dark brown dorsal zigzag pattern. The only naturally occurring medically important local snake is the nose-horned viper (*V. a. ammodytes*). Immediately after the bite he felt pain and noticed two puncture wounds on his finger. Within 10 min the left hand was entirely swollen. He called for help and walked to the nearest road. He was found sweating, faint and dizzy sitting by the road when the rescue team arrived 30 min later. The patient presented with oedema of the affected hand, extending up a third of the forearm toward the elbow. Upon arrival to the Emergency Department one hour after the viper bite, the patient was somnolent, bradycardic (55 beats/min) and normotensive (130/70 mmHg). He complained of intense pain at the envenomation site. Local oedema and erythema extended half way up the forearm. The remainder of his physical examination was unremarkable. Initial laboratory studies two hours after the bite revealed evidence of rhabdomyolysis (myoglobin 133 µg·L^−1^ (normal value: < 47 µg·L^−1^), creatine kinase 11.6 µkat·L^−1^ (normal value: < 2.85 µkat·L^−1^)), coagulopathy with an extended prothrombin time (0.63) and profound thrombocytopenia (20 × 10^9^ L^−1^). Pseudo-thrombocytopenia or analytical error due to possible *in vitro* formation of aggregates within a tube of the first blood sample was excluded by microscopic examination of blood smear and use of different buffers. Treatment was undertaken with 0.9% NaCl (100 mL·h^−1^), after blood pressure dropped to 100/50 mmHg. Electrocardiogram (ECG) revealed sinus bradycardia at 45 beats/min. The patient had no neurological deficits. Four hours after the bite pain, oedema, erythema and lymphangitis extended to the upper arm and the envenomation was graded as grade 2b [4]. The patient was given 4 mL of Viperfav^TM^ diluted in 250 mL of 0.9% NaCl within 60 min. This was followed by a second dose of 4 mL of Viperfav™ diluted in 250 mL of 0.9% NaCl. 15 min later ECG revealed sinus bradycardia of 30 beats/min with a junctional escape rhythm that persisted for one hour. The patient’s blood pressure remained 100/50 mmHg. Afterwards, the patient remained normotensive with a pulse between 55–70 beats/min. No additional treatment was required. Follow up studies six hours after the bite (immediately after the second antivenom infusion) revealed normalisation of platelet count (170 × 10^9^ L^−1^) (Figure 1) and slight coagulopathy with prolongation of prothrombin time (0.58), while rhabdomyolysis (myoglobin 84 µg·L^−1^; creatine kinase 6.8 µkat·L^−1^) improved. Fibrinogen level was normal (2.41 g·L^−1^; normal value: 1.8–3.5 g·L^−1^). The spreading of oedema and erythema stopped and pain had decreased. 24 h after the bite a second drop in the platelet count occurred, with an eventual nadir of 40 × 10^9^ L^−1^ between 72 to 120 h post-snakebite (Figure 1). Petechiae and ecchymosis appeared on the affected limb. Microscopic examination of the blood smear showed giant platelets without schistocytes (platelet aggregates are not possible to observe in a blood smear). Direct and indirect anti-platelet antibody tests were negative, as were direct and indirect Coombs tests. All the other laboratory results, including white and red blood cells, glucose, electrolytes, urea, creatinine, myoglobin, hepatocellular enzyme levels, lactate, gas blood analysis, coagulation studies, fibrinogen and D-dimer remained within normal limits (data not shown). On the fifth day the platelet count increased, finally reaching normal values on the eighth day. The patient was ultimately discharged in good condition.

### 2.2. Detection of V. a. ammodytes Venom in Sera Samples

Serum *V. a. ammodytes* venom level two hours after the bite was 129 ng·mL^−1^. Concentrations of *V. a. ammodytes* venom in subsequent sera samples are presented in Figure 1.

### 2.3. Pharmacokinetics of Antivenom Level Decrement

Pharmacokinetic parameters were derived from the serum antivenom concentration-time data fitted into a two-compartment model. The patient received two vials of Viperfav™ by intravenous infusion. A pre-treatment serum sample taken approximately 2 h after the snakebite contained no detectable antivenom, while those obtained after fabotherapy resembled a biexponential decline of concentration of F(ab’)_2_ fragments according to the following mathematical equation (Figure 1):

when *t* ≤ *t*_inf_,
  *c*(*t*) = *A ×* (*1* − e^−α*t*^) + *B* × (1 − e^−β*t*^)
when *t* > *t*_inf_,
  *c*(*t*) = *A* × (e^−α(*t* − *tinf*)^ – e^−α*t*^) + *B* × (e^−β(*t* − *tinf*)^ – e^−β*t*^)

where *c*(*t*) is the antivenom concentration in the serum at any given time *t*. Coefficient *B* is the *y*-intercept of the extrapolated line representing the elimination phase, while hybrid rate constant β is the slope. The *y*-intercept of the line was obtained by plotting the residuals—difference between the observed serum concentration-time data and the matching values on the extrapolated line versus their corresponding time values is equal to the coefficient *A* in the equation. The hybrid rate constant α is the slope.

Compartmental analysis revealed that distribution (*t*_1/2α_) and elimination half-lives (*t*_1/2β_) were 6.6 h and 97.6 h, respectively. The steady-state distribution volume (*V*_ss_) corresponded to 213.7 mL·kg^−1^. The mean residence time (MRT) was 130.3 h. The area under the antivenom concentration-time curve at *t* = ∞ (AUC_∞_) and the area under the first(-order) moment curve (AUMC) were determined as 6095 μg·h·mL^−1^ and 793,893 μg·h^2^·mL^−1^, respectively. The systemic clearance (CL) was 1.64 (mL·h^−1^)·kg^−1^ (Table 1).

## 3. Discussion

Insight into the metabolism of antivenom, from absorption to elimination, is necessary to optimise the management of snakebite envenomation. In an attempt to contribute a more complete understanding of antivenom clinical effectiveness and pharmacokinetic behaviour, we report for the first time on a patient bitten by *V. a. ammodytes* and treated with Viperfav™ in whom the pharmacokinetics of antivenom immunoglobulin F(ab’)_2_ fragments was performed.

Viperfav™ was administered four hours after the *V. a. ammodytes* bite when local signs extended to the upper arm in the presence of severe thrombocytopenia and bradycardia with low blood pressure. At that time the envenomation was deemed to be at least grade 2b according to modified Audebert’s clinical severity grading (max. grade is 3) and the decision to use two vials of Viperfav™ was taken [4]. This promptly reduced local extension, improved systemic symptoms and laboratory results (e.g., thrombocytopenia). The improvement was consistent with the reported effectiveness of Viperfav™ in the treatment of *V. berus* and *V. aspis*-envenomed patients [6]. However, the recent prospective study concerning the use of Viperfav™ for *V. aspis* and *V. berus* envenomation showed that all clinical symptoms and pathological laboratory results disappeared within 24 h after treatment with a single dose of Viperfav™, with no recurrence of clinical or laboratory abnormalities [6]. These results corroborated earlier data that repeat administration did not produce any additional benefit to the clinical course in terms of the persistent functional impairment, incidence of haematoma or hospital length of stay [3,4]. On contrary, in the patient bitten by *V. a. ammodytes* pronounced thrombocytopenia recurred 24 h after the incident despite timely treatment with the recommended dose of Viperfav™ and initial improvement in the clinical picture. Accordingly, it seems that the recommended protocol of polyvalent Viperfav™ for *V. aspis* and *V. berus* may be inadequate for the efficient treatment of *V. ammodytes* bites.

In order to address issues concerning its clinical effectiveness, pharmacokinetics on Viperfav™’s immunoglobulin F(ab’)_2_ fragments was performed. It was shown that in *V. a. ammodytes* bitten patient, Viperfav™ systemic clearance was 1.64 (mL·h^−1^)·kg^−1^ with distribution and elimination half-lives of 7 h and 4 days respectively (Table 1). A relative comparison of this result and its interpretation in the light of other studies carried out on the pharmacokinetics of antivenoms in envenomated human, especially those based on F(ab’)_2_ fragments as active principle [8,9,10], revealed that the pharmacokinetic properties of Viperfav™ were consistent with those obtained for GPO antivenom (Thai Government Pharmaceutical Organisation), pepsin-digested anti-*Calloselasma rhodostoma* serum, with the exception of distribution half-lives [9]. The discrepancy probably resulted from infrequent blood sampling in the initial period following the administration of Viperfav™ and consequent omission of the rapid first phase, which might lead to a falsely longer distribution half-life. In comparison to other antivenoms that have been pharmacokinetically characterised using whole immunoglobulin G molecules or fabotherapics, the values with respect to Viperfav™’s elimination half-life and systemic clearance are among the highest obtained [8,9,10,11,12,13,14]. Nevertheless, it seems that in this patient an exhaustion of the available F(ab’)_2_ fragments occurring before all the relevant venom components were disabled was the likely cause of the recurrent thrombocytopenia after the *V. a. ammodytes* bite, since decrease of the serum F(ab’)_2_ fragments concentration and platelet count correlated remarkably well. This implies that the second drop in platelets was caused by the reappearance of free components of *V. a. ammodytes* venom during the clearance of F(ab’)_2_ fragments. These components of *V. a. ammodytes* venom might be sufficient for haemostatic disturbances to recur because of their potent toxicity. Accordingly, a repeated dose of Viperfav™ in *V. a. ammodytes* envenomed patients should now be evaluated during the delayed phase of thrombocytopenia (despite the fact that Viperfav™’s elimination half-life and systemic clearance are amongst the highest). Rebound of venom antigenemia and an association with recurrent thrombocytopenia is yet to be examined, since in this case *V. a. ammodytes* venom detected during recurrent thrombocytopenia was most likely incorporated into soluble immune complexes with F(ab’)_2_ fragments.

In light of the recurrent phenomena demonstrated by the case we report here, it might be necessary for *V. a. ammodytes* envenomations to be treated with an additional dose(s) of Viperfav™. Higher doses of Viperfav™ could be associated with a higher risk of side effects, even though single dose treatment has been shown to be safe and without adverse reaction [6]. Interestingly in this patient, sinus bradycardia after *V. a. ammodytes* bite was aggravated and coupled with a junctional escape rhythm during the infusion of the second Viperfav™ vial, resolving only at the end of the infusion. Arrhythmias in *V. a. ammodytes* bitten patients could be also due to the *V. a. ammodytes* venom itself, particularly the ammodytoxins. These are capable of inducing several conduction and electrophysiological disturbances, including the occurrence of malignant ventricular arrhythmias [15], due to direct myo-cytotoxic, electrophysiological, prothrombotic and coronary vasoconstrictive effects [16,17].

## 4. Conclusions

The recommended single dose of polyvalent Viperfav™ for *V. aspis* and *V. berus* envenomations may be inadequate for snakebites by *V. a. ammodytes*. A second dose of Viperfav™ might be necessary despite its 97 h elimination half-life. Higher doses of Viperfav™ may be associated with a greater risk of arrhythmia or other adverse events. Closely monitoring patients during Viperfav™ infusion is absolutely prerequisite.

## 5. Materials and Methods

### 5.1. Quantification of V. a. ammodytes Venom in Sera Samples

Determination of *V. a. ammodytes* venom in sera samples was performed as follows. Microtiter plate was coated with in-house rabbit anti-*V. a. ammodytes* venom IgG (1 μg·mL^−1^) in 0.05 M carbonate buffer, pH 9.6 (100 μL/well) and left overnight at room temperature. After washing and blocking with 2% (*w*/*v*) BSA in PBS/T (0.05% (*w*/*v*) Tween 20 in PBS) buffer (200 μL/well) for 2 h at 37 °C, the investigated sera (2- or 4-fold diluted) were added in duplicates and incubated overnight at room temperature (RT). The whole venom solution (100 ng·mL^−1^) used as standard was prepared from pool of sera from healthy donors (25% or 50%, *v*/*v*) and added in eight serial 2-fold dilutions in duplicates (100 μL/well). The plate was extensively washed and incubated with in-house equine anti-*V. a. ammodytes* venom IgG (100 μL/well of 5.7 μg·mL^−1^) and then with horseradish peroxidase (HRP)-anti-equine IgG (100 μL/well of 4000-fold dilution). Incubation was performed for 2 h at 37 °C. Finally, after washing *o*-phenylenediamine (OPD) solution (5.5 mM in 0.15 M citrate-phosphate buffer, pH 5.0) with 30% H_2_O_2_ (0.5 μL/mL of OPD solution) was added and incubated for half an hour at RT in the dark. The enzymatic reaction was stopped with 12.5% H_2_SO_4_ (50 μL/well) and absorbance at 492 nm was measured.

### 5.2. Quantification of F(ab’)_2_ Fragments in Sera Samples

Sandwich enzyme-linked immunosorbent assay (ELISA) was developed as follows. Microtiter plate was coated with in-house rabbit anti-equine F(ab’)_2_ IgG (1 μg·mL^−1^) in 0.05 M carbonate buffer, pH 9.6 (100 μL/well) and left overnight at room temperature. After washing and blocking with 2% (*w*/*v*) BSA in PBS/T (0.05% (*w*/*v*) Tween 20 in PBS) buffer (200 μL/well) for 2 h at 37 °C, the investigated sera were added in a few different, as first determined, suitable dilutions in duplicates (100 μL/well). Viperfav™ (100 ng·mL^−1^) used as standard, was added in eight serial 2-fold dilutions (100 μL/well), also in duplicates. Incubation was performed overnight at RT. The plate was extensively washed and incubated with HRP-anti-equine IgG (100 μL/well of 5000-fold dilution) for 2 h at 37 °C. Finally, after washing OPD solution (5.5 mM in 0.15 M citrate-phosphate buffer, pH 5.0) with 30% H_2_O_2_ (0.5 μL/mL of OPD solution) was added and incubated for half an hour at RT in the dark. The enzymatic reaction was stopped with 1 M H_2_SO_4_ (50 μL/well) and absorbance at 492 nm was measured. Quantitative determination of F(ab’)_2_ content was done by multiplying each concentration obtained from the standard curve by the corresponding dilution factor. The assay was performed independently five times and the results are given as mean ± 95% confidence interval (CI).

### 5.3. Pharmacokinetic Analysis

Pharmacokinetic analysis of the measured concentrations was performed using PKSolver add-in software (version 2.0, China Pharmaceutical University, Nanjing, China) for Microsoft Excel [18]. Concentration-time data was fitted either to one-, two- or three-compartment model. Akaike information criterion (AIC) and Schwarz criteria (SC) were used for comparison of goodness of their fit [19].

## Figures and Tables

**Table 1:** Pharmacokinetic parameters of Viperfav™ in the patient envenomed by *V. a. ammodytes* after i.v. administration.

Pharmacokinetic Parameters	Values
*t*_1/2α_	6.56 h
*t*_1/2β_	97.64 h
*V*_ss_	213.7 mL·kg^−1^
MRT	130.25 h
AUC_∞_	6095 μg·h·mL^−1^
AUMC	793,893 μg·h^2^·mL^−1^
CL	1.64 (mL·h^−1^)·kg^−1^

*Legend: t*_1/2α_, distribution half-life; *t*_1/2β_, elimination half-life; *V*_ss_, steady-state volume of distribution; MRT, mean residence time; AUC_∞_, area under the curve at *t* = ∞; AUMC, area under the first(-order) moment curve; CL, systemic clearance.
